# Effects of time-restricted feeding and walking exercise on the physical health of female college students with hidden obesity: a randomized trial

**DOI:** 10.3389/fpubh.2023.1020887

**Published:** 2023-05-19

**Authors:** Haitao Liu, Shiying Chen, Haoyuan Ji, Zuanqin Dai

**Affiliations:** ^1^College of Physical Education, Henan University, Kaifeng, China; ^2^Research Center for Sports Reform and Development, Henan University, Kaifeng, China; ^3^Institute of Physical Fitness and Health, Henan University, Kaifeng, China

**Keywords:** time-restricted feeding, hidden obesity, female college students, body mass index, body fat, blood lipids, bone mineral density, walking exercise

## Abstract

**Purpose:**

Time-restricted feeding (TRF) is an emerging dietary pattern with many potential effects. This study focused on the effects of TRF and walking on the physical health of female college students with hidden obesity.

**Methods:**

A total of 77 female college students with hidden obesity, aged 18–22 years, were randomly assigned to a control group (CON, *N* = 19), time-restricted feeding group (TRF, *N* = 19), exercise group (EXE, *N* = 20), and TRF combined with exercise group (TRF + EXE, *N* = 19). The interventions lasted for 8 weeks. Tests assessing body shape, body composition, bone mineral density, blood lipid levels, and blood pressure were performed before and after the intervention.

**Results:**

(1) Intragroup comparison before and after the intervention revealed that the TRF, EXE, and TRF + EXE groups had significantly reduced body weight (*p* < 0.01), body mass index (BMI) (*p* < 0.05), and lean tissue mass (LTM) (*p* < 0.01) but increased total cholesterol (TC) levels (*p* < 0.05) after the intervention. Body fat percentage (BF%) increased considerably in the EXE and TRF + EXE groups (*p* < 0.01). (2) Post-intervention comparisons of body weight, BMI, LTM, adipose tissue mass (ATM), total bone mineral density (TBMD), blood lipid levels, and blood pressure between the intervention groups (TRF, EXE, and TRF + EXE) and the CON group showed no significant differences (*p* > 0.05). (3) A comparison of the changes between the groups before and after the intervention showed significant decreases in body weight in the TRF and TRF + EXE groups (*p* < 0.05) and in both BMI and LTM in the TRF, EXE, and TRF + EXE groups (*p* < 0.05) compared to those in the CON group. The BF% change in the EXE and TRF + EXE groups were significantly greater than that in the TRF group (*p* < 0.01).

**Conclusion:**

TRF effectively decreased body weight and BMI in female college students with hidden obesity. However, increased blood lipid levels and decreased LTM levels were also observed. The effects of TRF combined with exercise were not superior to those of TRF or walking alone in terms of body weight, body mass index, body composition, TBMD, or blood lipid levels. Therefore, TRF cannot be considered the best option for fat reduction in female college students with hidden obesity.

## 1. Introduction

Intermittent fasting (IF), which refers to a dietary intervention that alternates between free eating and fasting, has recently become a popular dietary strategy. Studies have shown that it has many potential benefits for the body (1–3). Recent research has demonstrated that IF can boost gut microbial metabolites and encourage the regeneration and functional repair of peripheral nerve axons (4). IF can be classified into (5) alternate day fasting (ADF), whole-day fasting (WDF), and time-restricted feeding (TRF). ADF usually refers to alternating between a “feast day,” i.e., a day of free eating, and a “fast day,” i.e., a day of reduced food intake (6). WDF refers to complete fasting or strict caloric restriction for 1–2 days per week (7). TRF involves food consumption restricted to specific times of the day, with the remaining periods being fasting periods (8). Although there are different forms of IF, it is a sustainable and easy-to-implement lifestyle, and each form has different effects on the body (9–16). Studies have shown that ADF has beneficial effects on the control of body weight, waist circumference, systolic blood pressure (SBP), and fasting blood glucose levels (17); however, the participants reported greater levels of hunger, which is not conducive to long-term compliance (18). The difference between TRF and other dietary interventions is that this dietary pattern restricts food intake to a specified time of the day with fasting outside of this period, does not restrict the type or quantity of food consumed or limit the consumption of water, is closer to normal eating patterns, and is easy to implement and adhere to over time without serious side effects (19, 20). The concept of TRF comes from the study of the body’s circadian rhythm and was first used to study the effects of the timing of food intake on the biological clock system (21). The main mechanism involves optimizing the body’s anabolism and catabolism by adjusting the timing of food intake, increasing the diversity of intestinal microorganisms, and maintaining homeostasis (19). Animal studies have shown that TRF intervention can prevent obesity and metabolic diseases (22), and improve cardiac function (23). Human studies have revealed that TRF not only reduces body weight but also improves body composition and metabolic health (20, 22), delays aging (24), and increases gut microbial diversity (19). Notably, some studies have reported that the benefits of TRF in humans are independent of food intake and weight loss (10). Therefore, some researchers believe that TRF has the potential to promote a healthy lifestyle (25).

Recently, there has been an increased scientific interest worldwide in hidden obesity. Hidden obesity is also known as normal-weight obesity (26, 27). It is a body state that precedes the emergence of obesity and manifests in individuals with a body mass index (BMI) in the normal range but with a higher body fat percentage (BF%) (28). The international community generally considers women with a BF% of 30% or more to be “obese” (29). Adult hidden obesity is often assessed based on a BMI ≥ 18.5 kg/m^2^ and ≤23.9 kg/m^2^ and BF% ≥ 30% (30, 31). Hidden obesity is an under-recognized but prevalent public health problem in Asian populations (32). The problem of hidden obesity is particularly prominent among women (31), and is almost non-existent in men (33). It has been reported that one-third of women aged over 19 years have “hidden obesity” (34). Compared with obesity, “hidden obesity” is easily ignored and therefore poses a greater risk to people’s health. This group has excess body fat, which increases the risk of cardiovascular and other chronic diseases such as insulin resistance, hypertension, and dyslipidemia (35, 36). New therapies are urgently needed because existing obesity intervention strategies are cumbersome and ineffective, and most are challenging to maintain over time. Recent studies have shown that TRF can reduce body weight, lower blood pressure and blood lipid levels, and promote metabolic health (19, 37, 38). TRF is anticipated to be a new way of life for those attempting to lose weight, because people do not need to keep track of their food intake or count calories while eating (25). The lack of calorie restriction in TRF has successfully attracted an increasing number of young obese people to try this diet (39). However, the effect of TRF on individuals with hidden obesity has not yet been elucidated.

This study aimed to evaluate the effects of TRF, walking exercise, and TRF combined with walking exercise in female college students with hidden obesity. The intervention also Additionally, we specifically aimed to explore the effects of TRF and exercise in women with hidden obesity.

## 2. Materials and methods

### 2.1. Participants

This study enrolled 90 female students aged 18–22 years from Henan University between September 2021 and April 2022. The inclusion criteria were BMI between 18.5 kg/m^2^ and 23.9 kg/m^2^, BF% ≥ 30% (40, 41), no significant weight fluctuation (weight gain or loss ≤5 kg) in the 3 months before the study, and no underlying diseases or contraindications to exercise. After screening, 10 individuals who did not meet the eligibility criteria were excluded. Finally, 80 individuals were included in the study. All the participants provided written informed consent. This study was approved by the Ethics Committee of Human Research of Henan University (HUSOM2021-210).

**Figure 1:**
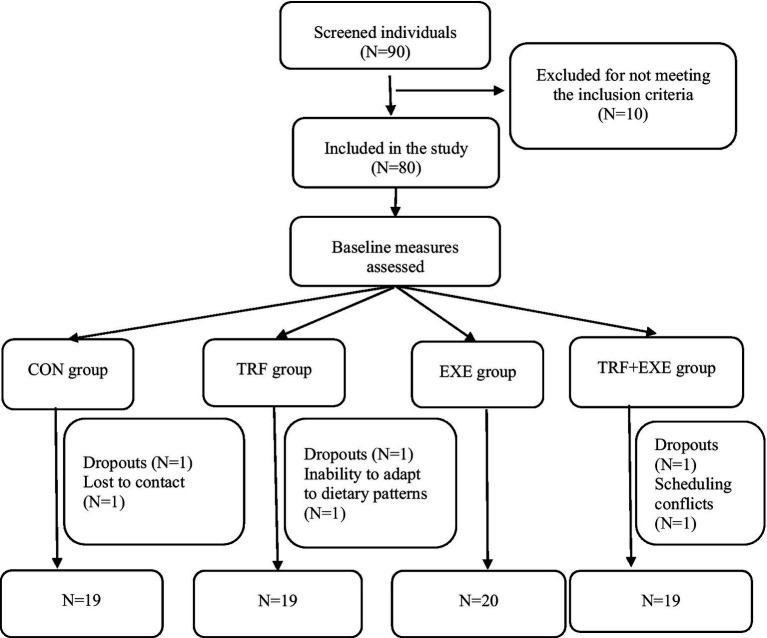
Flow of participant selection in the study. CON, control; TRF, time-restricted feeding; EXE, exercise; TRF + EXE, time-restricted feeding combined with exercise.

We used random number sequences for this group (25). The participants were assigned to four groups in a ratio of 1:1:1:1. The four groups were the control (CON, *N* = 20), TRF (*N* = 20), exercise (EXE, *N* = 20), and TRF combined with exercise (TRF + EXE, *N* = 20) groups. A total of 77 participants completed the study (completion rate, 96.25%) after 8 weeks of intervention, including 19 in the CON group (95.00%), 19 in the TRF group (95.00%), 20 in the EXE group (100%), and 19 in the TRF + EXE group (95.00%). Specifically, one participant withdrew midway for personal reasons, one participant was excluded because of incomplete post-intervention data collection, and one participant left because she could not adjust to the TRF diet strategy (Figure 1).

### 2.2. Study design

This was an 8 week (42), randomized, controlled, parallel-arm diet trial. During the intervention period, all the participants lived freely and could choose their own food. The CON group participants were asked to maintain their usual lifestyle (42). The TRF group followed TRF for 8 h (43), that is, eating at their discretion between 10:00 and 18:00 daily and fasting for the remainder of the day, without restricting the kinds of foods or the amount that they could consume, and without any specific dietary guidelines. According to the physical activity classification criteria of Tudor-Locke et al. (44), 10,000–12,499 steps per day are considered physically active. Participants in the TRF + EXE group were required to follow 8 h of TRF and achieve 11,000–12,499 steps per day. All the participants wore triaxial accelerometers to monitor their daily walking activities.

### 2.3. Indicator testing

#### 2.3.1. Height measurement

The height of the participants was measured by members of the study team. The participants were asked to stand barefoot in an upright position with their heels and forefeet at a 60°angle on a height test meter. The height reading was accurately measured to the nearest 0.01 cm, and the average of three measurements was calculated.

#### 2.3.2. Body weight, body composition, and bone mineral density measurements

Dual-energy X-ray absorptiometry (Hologic, Horizon-Wi, United States) was used to evaluate body weight, BF%, adipose tissue mass (ATM), lean tissue mass (LTM), and total bone mineral density (TBMD). A quality assurance procedure was performed prior to testing. The participants were asked to remove any metal objects (such as belts, keys, or cell phones) and lie flat on the testbed with their feet turned inward for scanning. The entire test process took approximately 15 min, and the participants were asked to remain relaxed and avoid moving as much as possible during the test to avoid affecting the test results.

#### 2.3.3. Blood lipid index data

One day before the start and 1 day after the end of the intervention, early morning fasting venous blood samples were collected from all participants in a standardized manner at the First Affiliated Hospital of Henan University. Total cholesterol (TC), triglyceride (TG), high-density lipoprotein cholesterol (HDL-C), and low-density lipoprotein cholesterol (LDL-C) levels were measured.

#### 2.3.4. Blood pressure measurement

Systolic blood pressure (SBP) and diastolic blood pressure (DBP) were measured using medical automatic electronic blood pressure monitor (Omron, HBP-9020, Japan). Participants were asked to avoid strenuous activity for 1 h before measurement and to rest quietly for 10 min before measurement. The right forearm was placed flat on a table with the palm facing up while the participant was seated, and the pulse pressure band was fastened to the participant’s upper arm with an appropriate amount of tension. The same upper arm was used to record three measurements and the average SBP and DBP readings were recorded as the final blood pressure.

### 2.4. Walking monitoring

An ActiGraph GT3X triaxial accelerometer was used to monitor the participants’ daily step count and energy expenditure. The participants were instructed to wear the accelerometer on their left wrist. Table 1 shows the average daily energy expenditure and steps taken during the 8 week intervention.

**Table 1:** Daily steps during the 8 week intervention.

Group	Daily steps
CON	6427.69 ± 1906.47
TRF	6830.93 ± 1716.86
EXE	11506.7 ± 1241.59
TRF + EXE	12076.9 ± 1482.28

CON, control; TRF, time-restricted feeding; EXE, walking exercise; TRF + EXE, time-restricted feeding combined with walking exercise.

### 2.5. Quality control

To ensure that each participant could apply the TRF approach and walking exercise program independently, a thorough description of both activities was provided to all participants prior to trial commencement. During the intervention, weekly communication was conducted with the participants, and any adverse reactions experienced by the intervention groups were recorded. Participants who experienced serious adverse reactions were asked to discontinue participation. Simultaneously, the research team supervised the intervention by asking the participants to upload their diet and exercise data daily to the WeChat platform to promptly detect problems and provide targeted assistance.

### 2.6. Sample size

We used G* Power software (version 3.1.9.7) to calculate the sample size. At least 76 participants were required to achieve a statistical power of 90% at the *α* = 0.05.

### 2.7. Statistical analysis

Experimental data are expressed as means ± standard deviation. The data were tested for normality. One-way ANOVA was used to test for homogeneity between the groups before the intervention and to detect differences in the changes between before and after the intervention. *Post hoc* multiple comparisons were performed using the least significant difference test. Paired *t*-tests were used for intragroup comparisons before and after the intervention. Statistical significance was set at *p* < 0.05 or *p* < 0.01.

## 3. Results

### 3.1. Baseline characteristics

No statistically significant differences were found in the participant preintervention baseline indicators among the CON, TRF, EXE, and TRF + EXE groups (Table 2).

**Table 2:** Baseline characteristics of the study participants (*N* = 77).

Index	CON group (*N* = 19)	TRF group (*N* = 19)	EXE group (*N* = 20)	TRF + EXE group (*N* = 19)
Age (years)	20.08 ± 1.76	20.29 ± 1.79	20.09 ± 1.35	19.93 ± 0.61
Height (cm)	164.31 ± 5.47	163.00 ± 4.73	160.68 ± 6.37	161.00 ± 5.21
Weight (kg)	54.14 ± 5.81	56.34 ± 4.70	53.62 ± 5.20	55.32 ± 4.43
BMI (kg/m^2^)	20.32 ± 1.06	21.63 ± 1.24	21.35 ± 1.56	21.86 ± 1.48
BF%	37.35 ± 3.85	38.21 ± 3.32	38.32 ± 2.62	39.08 ± 2.31
ATM (kg)	20.35 ± 38.25	21.60 ± 30.99	20.58 ± 27.74	21.64 ± 24.65
LTM (kg)	33.79 ± 28.01	34.74 ± 26.18	33.04 ± 31.05	33.67 ± 25.47
TBMD (g/cm^2^)	1.07 ± 0.06	1.08 ± 0.07	1.06 ± 0.08	1.05 ± 0.06
TC (mmol/L)	3.93 ± 0.70	3.91 ± 0.36	3.86 ± 0.39	4.00 ± 0.64
TG (mmol/L)	0.91 ± 0.51	0.80 ± 0.25	0.87 ± 0.30	0.85 ± 0.41
HDL-C (mmol/L)	1.45 ± 0.20	1.38 ± 0.19	1.42 ± 0.24	1.44 ± 0.18
LDL-C (mmol/L)	2.25 ± 0.74	1.98 ± 0.43	2.11 ± 0.65	2.45 ± 0.69
DBP (mmHg)	73.47 ± 11.65	71 ± 7.43	75.71 ± 8.27	70.62 ± 8.63
SBP (mmHg)	111.71 ± 4.972	110 ± 8.23	113.18 ± 9.857	113 ± 8.866

The data were expressed as means ± standard deviations. CON, control; TRF, time-restricted feeding; EXE, walking exercise; TRF + EXE, time-restricted feeding combined with walking exercise; BMI, body mass index; BF%, body fat percentage; ATM, adipose tissue mass; LTM, lean tissue mass; TBMD, total bone mineral density; TC, total cholesterol; TG, triglyceride; HDL-C, high-density lipoprotein cholesterol; LDL-C, low-density lipoprotein cholesterol; DBP, diastolic blood pressure; SBP, systolic blood pressure.

### 3.2. Changes in body weight and body mass index

After the 8 week intervention, intragroup comparisons revealed that participants in the TRF, EXE, and TRF + EXE groups showed significant reductions in both weight and BMI (*p* < 0.01). Moreover, the mean reduction in weight from the baseline was 3.03 kg, 1.96 kg, and 2.52 kg in the TRF, EXE, and TRF + EXE groups, respectively (Table 3).

**Table 3:** Changes in the health indicators of the participants before and after intervention.

Index	CON (*N* = 19)				TRF (*N* = 19)				EXE (*N* = 20)				TRF + EXE (*N* = 19)			
	Pre-intervention	Post-intervention	*T* value	*P* value	Pre-intervention	Post-intervention	*T* value	*P* value	Pre-intervention	Post-intervention	*T* value	*P* value	Pre-intervention	Post-intervention	*T* value	*P* value
Weight (kg)	54.14 ± 5.81	53.40 ± 5.53	2.03	0.082	56.34 ± 4.70	53.31 ± 4.38**	8.15	0.000	53.62 ± 5.20	51.66 ± 5.52**	5.44	0.000	55.32 ± 4.43	52.79 ± 4.88**	5.41	0.000
BMI (kg/m^2^)	20.32 ± 1.06	19.74 ± 1.22**	4.52	0.003	21.63 ± 1.24	20.05 ± 1.22**	10.85	0.000	21.35 ± 1.56	20.00 ± 1.63**	10.14	0.000	21.86 ± 1.48	20.39 ± 1.90**	7.84	0.000
BF%	37.35 ± 3.85	39.87 ± 4.96**	−3.85	0.006	38.21 ± 3.32	38.54 ± 3.10	−0.72	0.482	38.32 ± 2.62	41.64 ± 2.56**	−8.44	0.000	39.08 ± 2.31	42.06 ± 2.85**	−6.25	0.000
ATM (kg)	20.35 ± 38.25	20.74 ± 45.68	−1.28	0.24	21.60 ± 30.99	21.34 ± 25.83	0.72	0.483	20.58 ± 27.74	20.89 ± 30.94	−1.26	0.224	21.64 ± 24.65	21.44 ± 29.18	0.91	0.375
LTM (kg)	33.79 ± 28.01	32.66 ± 22.76	2.34	0.052	34.74 ± 26.18	31.97 ± 28.89**	11.28	0.000	33.04 ± 31.05	30.77 ± 30.57**	10.56	0.000	33.67 ± 25.47	31.35 ± 25.16**	6.25	0.000
TBMD (g/cm^2^)	1.07 ± 0.06	1.08 ± 0.07	−2.03	0.059	1.08 ± 0.07	1.08 ± 0.06	0.14	0.892	1.06 ± 0.08	1.07 ± 0.08	−1.46	0.160	1.05 ± 0.06	1.05 ± 0.05	−0.23	0.820
TC (mmol/L)	3.93 ± 0.70	4.23 ± 0.67*	−2.86	0.011	3.91 ± 0.36	4.38 ± 0.55**	−3.12	0.006	3.86 ± 0.39	4.07 ± 0.50*	−2.16	0.044	4.00 ± 0.64	4.40 ± 0.74**	−2.9	0.010
TG (mmol/L)	0.91 ± 0.51	0.97 ± 0.39	−1.04	0.314	0.80 ± 0.25	1.06 ± 0.37*	−2.17	0.045	0.87 ± 0.30	0.99 ± 0.41	−1.37	0.188	0.85 ± 0.41	0.95 ± 0.41	−1.04	0.311
HDL-C (mmol/L)	1.45 ± 0.20	1.62 ± 0.41	−1.89	0.077	1.38 ± 0.19	1.59 ± 0.35	−1.96	0.067	1.42 ± 0.24	1.49 ± 0.26	−0.96	0.350	1.44 ± 0.18	1.64 ± 0.24**	−4.8	0.000
LDL-C (mmol/L)	2.25 ± 0.74	2.39 ± 0.77	−1.26	0.225	1.98 ± 0.43	2.44 ± 0.71*	−2.66	0.017	2.11 ± 0.65	2.32 ± 0.46	−1.38	0.184	2.45 ± 0.69	2.72 ± 0.69*	−2.43	0.026
DBP (mmHg)	73.47 ± 11.65	74.36 ± 11.46	−0.71	0.491	71.00 ± 7.43	69.40 ± 9.42	0.54	0.598	75.71 ± 8.27	74.28 ± 10.53	0.48	0.635	70.62 ± 8.63	69.09 ± 10.78	0.91	0.372
SBP (mmHg)	111.71 ± 4.97	112.8 ± 4.43	−1.00	0.33	110.00 ± 8.23	113.11 ± 4.61	−1.75	0.101	113.18 ± 9.86	109.02 ± 4.44	1.67	0.114	113.00 ± 8.87	110.71 ± 6.47	0.93	0.362

In the intra-group comparison, * indicates *P* < 0.05, and ** indicates *P* < 0.01. CON, control; TRF, time-restricted feeding; EXE, walking exercise; TRF + EXE, time-restricted feeding combined with walking exercise; BMI, body mass index; BF%, body fat percentage; ATM, adipose tissue mass; LTM, lean tissue mass; TBMD, total bone mineral density; TC, total cholesterol; TG, triglyceride; HDL-C, high-density lipoprotein cholesterol; LDL-C, low-density lipoprotein cholesterol; DBP, diastolic blood pressure; SBP, systolic blood pressure.

**Figure 2:**
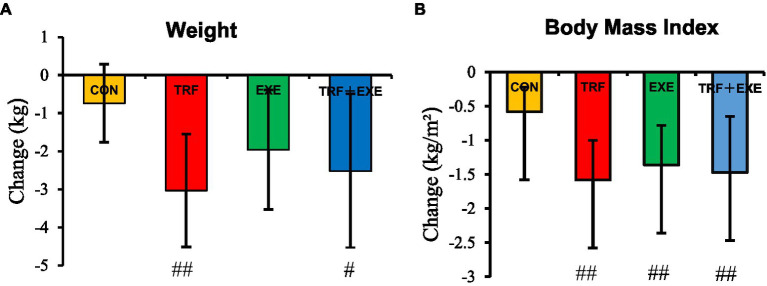
Changes in body weight and body mass index after the 8 week intervention. The changes were expressed as means ± standard error and were calculated by subtracting the 8 week value from the baseline value. **(A)** Changes in body weight and **(B)** changes in body mass index. Compared with the CON group, # indicates *p* < 0.05, ## indicates *p* < 0.01. CON, control; TRF, time-restricted feeding; EXE, walking exercise; TRF + EXE, time-restricted feeding combined with walking exercise.

After the 8 week intervention, intergroup comparisons revealed that the change in weight was significantly higher in the TRF (*p* < 0.01) and TRF + EXE (*p* < 0.05) groups than in the CON group, but there was no significant difference among the three intervention groups (*p* > 0.05) (Figure 2A). Compared to the CON group, changes in BMI decreased significantly in the TRF, EXE, and TRF + EXE groups (*p* < 0.01) (Figure 2B).

### 3.3. Changes in body composition and bone mineral density

After the 8 week intervention, the intragroup comparison revealed that the BF% increased significantly in the EXE and TRF + EXE groups (*p* < 0.01). LTM decreased significantly in the TRF, EXE, and TRF + EXE groups (*p* < 0.01), but there was no significant change in ATM or TBMD (*p* > 0.05) (Table 3).

**Figure 3:**
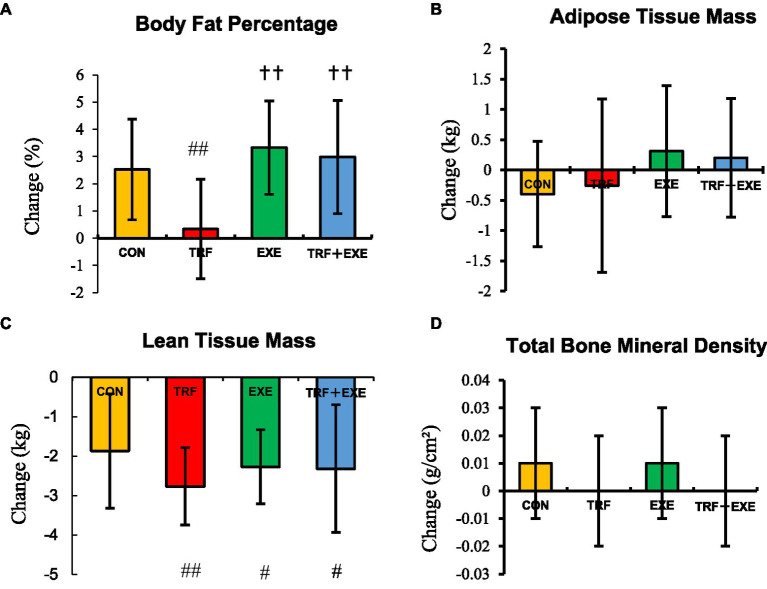
Changes in the body composition and total bone mineral density after the 8 week intervention. Changes are expressed as means ± standard errors and calculated by subtracting the 8 week value from the baseline value. **(A)** Change in body fat percentage, **(B)** adipose tissue mass, **(C)** lean tissue mass, and **(D)** total bone mineral density. Compared with the CON group, # indicates *p* < 0.05, ## indicates *p* < 0.01. Compared with the TRF group, † *p* < 0.05 and †† indicates *p* < 0.01. CON, control; TRF, time-restricted feeding; EXE, walking exercise; TRF + EXE, time-restricted feeding combined with walking exercise.

After the 8 week intervention, BF% showed a positive change in all groups, but the TRF group showed the smallest change (*p* < 0.01) (Figure 3A). The change in BF% was significantly greater in the EXE and TRF + EXE groups than in the TRF group (*p* < 0.01) (Figure 3A). The change in LTM was significantly smaller in the three intervention groups than in the CON group (*p* < 0.05) (Figure 3C). There were no significant differences in the changes in ATM and TBMD among the participants in all four groups (*p* > 0.05) (Figures 3B,D).

### 3.4. Changes in blood lipid levels and blood pressure

After the 8 week intervention, the intragroup comparison revealed that TC levels increased significantly in the TRF, EXE, and TRF + EXE groups (*p* < 0.05), whereas DBP and SBP did not change significantly (*p* > 0.05). TG levels increased significantly in the TRF group (*p* < 0.05), HDL-C levels increased significantly in the TRF + EXE group (*p* < 0.01), and LDL-C levels increased significantly in both TRF and TRF + EXE groups (*p* < 0.05) (Table 3).

**Figure 4:**
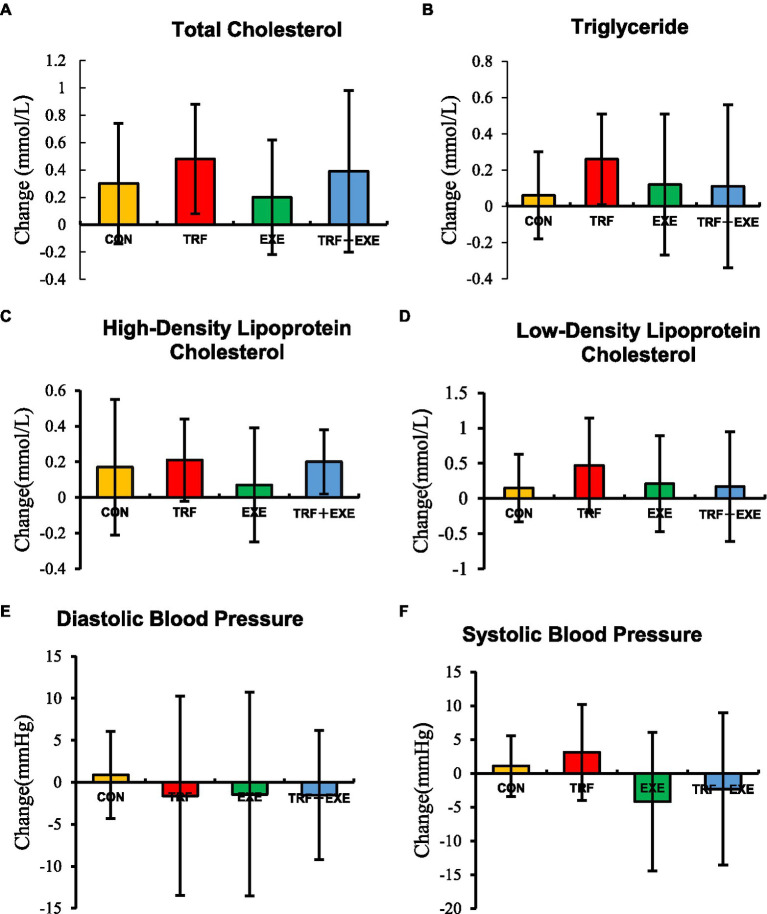
Changes in the blood lipid levels and blood pressure after the 8 week intervention. Changes are expressed as means ± standard errors and calculated by subtracting the 8 week value from the baseline value. **(A)** Change in total cholesterol levels, **(B)** triglyceride levels, **(C)** high-density lipoprotein cholesterol levels, **(D)** low-density lipoprotein cholesterol levels, **(E)** diastolic blood pressure, and **(F)** systolic blood pressure. CON, control; TRF, time-restricted feeding; EXE, walking exercise; TRF + EXE, time-restricted feeding combined with walking exercise.

As shown in Figure 4, there were no significant differences in the changes in blood lipid levels or blood pressure among the participants in all four groups (*p* > 0.05).

### 3.5. Adverse reactions

No serious adverse reactions were reported in the participants in the three intervention groups; however, the number of participants experiencing minor adverse reactions was higher in the TRF (57.9% of participants), and TRF + EXE (78.9%) groups than in the EXE group (40%) (Table 4). Notably, one participant in the TRF + EXE group experienced strong hunger in the evening, and her eating time changed to eating between 12:00 and 20:00. However, she was unable to concentrate in class. Eventually, the participant was unable to continue with TRF and decided to discontinue participation in the experiment.

**Table 4:** Adverse reactions of the participants during the intervention.

Adverse reactions	TRF group		EXE group		TRF + EXE group	
	Number of participants	Incidence of adverse reactions (%)	Number of participants	Incidence of adverse reactions (%)	Number of participants	Incidence of adverse reactions (%)
None	8	42.1	12	60.0	4	21.1
Lack of concentration	5	26.3	0	0.0	6	31.6
Dizzy	1	5.3	0	0.0	2	10.5
Tired	2	10.5	2	10.0	1	5.3
Thirsty	1	5.3	0	0.0	0	0.0
Irritable	2	10.5	0	0.0	1	5.3
Leg pain	0	0.0	3	15.0	2	10.5
Back pain	0	0.0	3	15.0	3	15.8

TRF, time-restricted feeding; EXE, walking exercise; TRF + EXE, time-restricted feeding combined with walking exercise.

## 4. Discussion

Obesity is a global public health issue that is attracting widespread public attention. The TRF dietary pattern allowed participants to reduce their energy intake (45), resulting in weight loss (25). The results of this study revealed that 8 weeks of TRF reduced body weight and BMI but caused an increase in blood lipid levels and a decrease in lean tissue mass in female college students with hidden obesity. No advantages were observed over 8 weeks in the TRF + EXE group compared to the TRF or EXE alone groups in terms of body weight, BMI, body composition, bone mineral density index, and blood lipid levels.

### 4.1. Effect of time-restricted feeding and walking exercise on body composition of hidden obesity in female college students

This study found that after the 8 week intervention, the BF% of the participants in the TRF and TRF + EXE groups increased considerably; in the EXE and TRF + EXE groups, LTM decreased significantly after the 8 week intervention. These results indicate that among female college students with hidden obesity, the 8 week TRF and walking exercise intervention had a negative impact on BF% and LTM. Although studies have shown that TRF has many health benefits, its effects on weight loss in humans remain controversial (46). Experimental animal studies have found that although TRF can reduce body weight, it can also lead to increased body fat and skeletal muscle loss (47). IF was found to be associated with a significant reduction in skeletal muscle mass. Experimental animal studies have shown that IF reduces body weight but leads to increased BF% and muscle loss. In human studies, the TRF dietary pattern was associated with decreased LTM (25, 48, 49). Another study found that subjects in both the 4 h TRF group and 6 h TRF groups showed a significant decrease in lean muscle tissue over 8 weeks compared to the normal diet group (25), and loss of muscle mass was associated with protein synthesis and catabolism (49). An inhibitory effect of TRF on LTM increase has also been reported (50). The results of the present study suggest that TRF and walking exercise reduced the muscle mass of the participants.

Another reason for the results of this study may be attributable to the eating behaviors of college students. Eating behavior is an important factor influencing the development of hidden obesity (36). LTM is a reflection of an individual’s muscle mass (51). Skeletal muscle mass is largely influenced by nutritional status and physical activity (47). A correlation between lower LTM and the eating habits of college students at school has been previously reported (52). This may also be because dietary factors play a significant role in the development of hidden obesity (36). The daily diet of the participants typically included meals from the school cafeteria or the consumption of streetside snacks, which mainly comprised fried foods, according to a preliminary understanding of the diet and exercise habits of female college students with hidden obesity. Therefore, eating habits in the sense of the type of food consumed must be carefully considered when evaluating the effects of TRF (53).

Additionally, walking is a low-intensity and prolonged aerobic activity (54) that improves the physical condition only slightly and has no influence on the ability to grow muscles in a short period. Studies have found that obese men and women who walked for 35–45 min at a moderate pace 3–5 times per week showed a reduction in the muscular cross-sectional area with no positive effects on the muscle mass (55). Walking is the most common form of outdoor activity in life, does not cause serious muscle and bone damage, does not require specific venues and equipment, and is a safe, economical, and simple form of exercise (56, 57). Walking 10,000 steps per day is considered an alternative to physical activity for public health and is comparable to 150 min of moderate-intensity physical activity per week. In this study, the energy expenditure of the EXE group increased while the same food intake was maintained, leading to a negative energy balance. Together, exercise and negative energy balance caused a decrease in LTM (58). Therefore, form a public health perspective, it is important to consider guidelines regarding exercise form and intensity when recommending exercise.

It is important to note that even though the weight of the participants in the intervention groups showed a declining trend, the reduction in LTM was the main reason for weight loss caused by TRF, resulting in an increased BF% in the subjects. Therefore, measures are required to compensate for this effect on the LTM. The effect of resistance exercise on reducing BF% and increasing LTM in participants should be properly considered (59). Lifestyle interventions are essential for increasing LTM and reducing ATM (60). The two main factors that influenced student BF% were diet and PA. Body composition is influenced by several variables including dietary intake and exercise. Scientifically proven effective exercise and a sensible food and nutrition plan are necessary for a fit physique, and these factors working synergistically have the greatest potential to enhance physical health (61).

### 4.2. Effect of time-restricted feeding and walking exercise on BMD of hidden obesity in female college students

In this study, there were no significant differences in TBMD between the TRF, EXE, and TRF + EXE groups before and after the intervention. Increased fat and reduced muscle mass are characteristics of individuals with hidden obesity. Excess fat also has a negative impact on bone mineral density (62) and may damage human bone health (63), whereas maintaining muscle health helps improve bone density. IF regimens have been found to be beneficial to human health in some studies; however, their effects on bone health remain unknown. One study reported that IF damages bone (64). Exercise and dietary habits have an important impact on human BMD, and positive exercise and dietary habits improve BMD. Healthy dietary habits allow the body to secure essential substances for bone synthesis, such as proteins, which, combined with the benefits of exercise, can promote higher BMD. Exercise is another factor that affects BMD, and individuals who actively exercise have higher BMD levels than those who do not (65). The results of the present study suggest that the TRF and walking exercise interventions had no significant effect on participant BMD. It is likely that the effect on BMD was not observed because of the short duration of the intervention. Walking is a convenient activity to preserve BMD and prevent its reduction (66, 67). However, the intensity of the exercise in this study was not sufficiently high to increase participant BMD.

### 4.3. Effect of time-restricted feeding and walking exercise on blood lipids of hidden obesity in female college students

This study found that after the 8 week intervention, participants in different intervention groups showed varying degrees of elevated blood lipid levels. This suggests that the study intervention had a negative effect on lipid levels. To date, it has not been conclusively demonstrated whether TRF lowers blood lipid levels in humans. According to several studies, TRF simply modifies the timing of food intake and does not change the diet composition; hence, such eating practices can result in abnormal blood lipid levels (68). Additionally, compared to an eating routine, TRF did not affect any of the pertinent metabolic markers in a previous study (43). Current improvements in economic levels have resulted in a substantial change to diet structure with increased consumption of meat and evidence indicates that excessive intake of meat can lead to dyslipidemia (69). Moreover, individuals with hidden obesity have higher fat content, and their lifestyle habits increase the risk of dyslipidemia. Energy-balanced dietary patterns can effectively improve body weight, BF%, and blood lipid levels in overweight/obese individuals (70). Healthy food strategies may significantly improve blood lipid levels. Randomized controlled trials with large sample sizes are warranted to conclusively establish the effect of TRF on lipid levels in future studies.

## 5. Limitations

This study presents some limitations. First, the sample size was small and the intervention period was short. Further studies should be conducted involving large samples and over long periods of time. Second, relevant indicators were not tested for the 4 week intervention outcomes. Moreover, some indicators, such as blood glucose and fasting insulin levels, were not tested. A greater understanding of time-restricted eating patterns is required, including the optimal duration and frequency of fasting, long-term compliance, and their effect on reducing various diseases. Furthermore, in view of the heterogenous findings reported to date, the effects of time-restricted eating in different healthy populations needs to be clarified in future studies.

## 6. Conclusion

Although TRF reduced body weight and BMI in the participants of this study, it also resulted in an increase in blood lipid levels and loss of LTM. In terms of body weight, BMI, body composition, TBMD, and blood lipid levels, TRF combined with walking exercise did not achieve better results than TRF or walking exercise alone. Therefore, TRF cannot be considered as the best option for fat reduction and obesity prevention in female college students with hidden obesity.

## Data availability statement

The original contributions presented in the study are included in the article/supplementary material, further inquiries can be directed to the corresponding author.

## Ethics statement

The studies involving human participants were reviewed and approved by This project was approved by the Ethics Committee of Human Research of Henan university (HUSOM2021-210). The patients/participants provided their written informed consent to participate in this study.

## Author contributions

HL: conceptualization, validation, writing—review and editing, supervision, and project administration. HL and SC: methodology, formal analysis, and writing—original draft preparation. SC and HJ: software. SC, HJ, and ZD: investigation. ZD and SC: data curation. SC: visualization. All authors contributed to the article and approved the submitted version.

## Funding

This research was funded by the Henan Science and Technology Development Project (grant number 202102310320), the Kaifeng Science and Technology Development Project (grant number 2003006), the Postgraduate Cultivating Innovation and Quality Improvement Action Plan of Henan University (grant number SYL-AL2022011), and the Henan University Teaching Reform Project (grant number HDXJJG2021-152).

## Conflict of interest

The authors declare that the research was conducted in the absence of any commercial or financial relationships that could be construed as a potential conflict of interest.

## Publisher’s note

All claims expressed in this article are solely those of the authors and do not necessarily represent those of their affiliated organizations, or those of the publisher, the editors and the reviewers. Any product that may be evaluated in this article, or claim that may be made by its manufacturer, is not guaranteed or endorsed by the publisher.
